# A Marketing Approach to a Psychological Problem: Problematic Smartphone Use on Adolescents

**DOI:** 10.3390/ijerph17072471

**Published:** 2020-04-04

**Authors:** Adnan Veysel Ertemel, Ela Ari

**Affiliations:** 1 Istanbul Commerce University, Faculty of Business Administration, International Trade Department, 34445 Istanbul, Turkey; 2 Istanbul Medipol University, Faculty of Humanities and Social Sciences, Psychology Department, 34810 Istanbul, Turkey; eari@medipol.edu.tr

**Keywords:** problematic smartphone use, adolescence, marketing, unhook, gamification

## Abstract

*Background:* Smartphones have become an indispensable part of the daily lives of adolescents in the 21st century, which is characterized by a highly digitized modern world. Besides their many advantages, smartphones might pave the way to compulsive usage and addictive experiences. To remedy this problem, this study proposes an authentic approach which integrates consumer behavior theories and techniques such as unhook and gamification. An education program has been designed based on these approaches to decrease the problematic smartphone use. *Method:* The participants of the education program consisted of 305 students (48.2% girls and 51.8% boys) with a mean age of 14.57 (SD = 0.74). The Demographic Form and Smartphone Addiction Scale for Adolescents (SASA) were conducted before the education program and three weeks after the education. *Results:* The results of the paired sample *t*-test analysis before and after the education program revealed that the SASA total scores decreased significantly (*p* < 0.01). There are significant differences in terms of gender, mothers’ education and class levels. *Conclusion:* This research emphasizes the role of an interdisciplinary approach to the addiction problem. The content used in the education program includes strategies that originally aimed at increasing consumption. The effectiveness of the program can be enhanced further in the future along with self-regulatory additions.

## 1. Introduction

With more than 3.1 [1] billion worldwide users as of 2019, which is expected to reach 3.8 billion by 2021 [2], smartphones have permanently changed our daily routines. They have become the de facto means for communication, socialization and access to information; and hence become inseparable in our lives [3]. When separated, individuals may encounter the following incidents: physiological withdrawal-like symptoms [4], increased anxiety [5] and even phantom vibration syndrome which can be described as the sensation that one’s smartphone is vibrating even though there is no notification coming [6], a syndrome experienced by the majority of users [7].

Not surprisingly, problematic smartphone use, which can be categorized as a form of technological addiction [8], has become a major global concern across societies [3]. Technology addiction may be characterized as a non-chemical behavioral addiction involving human-computer interaction [9]. On the other hand, problematic smartphone use, which is also interchangeably called as smartphone addiction or smartphone use disorder [10], can be defined as the overuse of smartphones in a manner that is difficult to control and has harmful effects on the other areas of life [11]. 

As smartphones are devices that can be carried anywhere and are available anytime, problematic smartphone use causes various serious potential problems. Among those problems pointed in the literature are their negative effect in interpersonal relations, [12], personal health [13] well-being [14,15], including decreased academic performance and decreased physical activity [16] as well as depression symptoms [17]. Validating this phenomenon, American Psychiatric Association addressed this behavior in Diagnostic and Statistical Manual of Mental Disorders-5 (DSM-5) by introducing the non-substance addiction as a psychiatric diagnosis, although there are still controversies on the topic [18].

Contemporary research on behavioral psychology points out that addictions arise out of problematic habit-driven behavior, which is a natural outcome of ineffective self-regulation and self-control [19]. As such, this repetitive habitual behavior increases the risk of personal and social problems [20]. Frequent interruptions were found to result in loss of productivity [21] and higher tendency of attention deficit hyperactivity disorder [22]. Even the mere existence of a smartphone on a table when studying was found to result in reduced cognitive capacity and up to a 10% decrease in IQ [23,24]. Studies show that it takes 23 min on average for a fully concentrated individual to come back to the previous state after being interrupted with a notification message [25]. Worsening the case, the probability that the individual will self-interrupt increases by 8%. 

As devices are being carried around all the time, Oulasvirta et al. [26] distinguish smartphone usage behavior as a series of ‘short duration, isolated, reward-based’ (SIRB) sessions as opposed to other technological devices like tablets and laptops. In this sense, informational rewards reinforce the checking behavior ultimately, performing it out of habit. As smartphones give access to a broad variety of sources for seeking information, socializing and entertainment, the overall reward value of ‘‘checking’’ habits increases as well.

Until recent times, severe criticisms of technology platforms due to their addictive-by-design experiences have gone unnoticed. However, the last couple of years witness a change in this case. As a result of the increasing awareness of these addictive-by-design techniques and the lobbying activities, technology platforms have started to adopt a more responsible code of conduct. As an example, Instagram and some other social media platforms changed their infinite scroll feature so that the users can scroll back up to two days. Again, as of 2019, Instagram started to hide the number of ‘likes’ to a post to avoid a meaningless competition of having more likes. 

Problematic smartphone use can be attributed to many factors. In the last decade, however, it is argued that digital platforms’ architected, finely tuned experiences that are designed to be addictive to deliberately make consumers spend prolonged periods of time on screens result in more problematic smartphone use [27,28,29,30,31]. Therefore, as opposed to looking from a mere psychological perspective, the need to adopt a multidisciplinary approach to prevent problematic smartphone use is brought into consideration.

Looking from a marketing perspective, we propose that using the same techniques that increase consumption—but in reverse manner—can effectively work in preventing problematic smartphone use. In the remainder of the paper, the defining characteristics of the modern digital marketing environment are discussed first. Afterwards, the mechanics of deliberately designed habit-forming experiences are explored. Finally, a consciousness-based preventive educational program on adolescents is proposed. The results of the study conducted among 305 students in 14 schools located in Istanbul, Turkey are also analyzed and discussed.

Adolescents were chosen as the target group since their acquaintance with smartphones is relatively new as opposed to their older counterparts, which implies that their smartphone behavior is not ossified yet. A study conducted late in 2019 in Turkey revealed that the most problematic smartphone use is seen in young adolescents and this problematic behavior rate decreases as the age of the users increases [32]. Another study conducted in Turkey published that 90.7% of the youngest age group (16–24). Internet use is listed as the heaviest compared to all other age groups. This group partially includes adolescents (10–19 years) which implies that this age group is the most susceptible to problematic smartphone use [33].

A recent global mobile consumer survey conducted in 28 countries across six continents revealed that Turkish consumers rank the highest in hourly checking of social networks and also highest in playing mobile games. Turkish consumers also ranked highest (26%) indicating physical health problems related with heavy smartphone usage [32]. These figures justify that adolescents are more prone to the risks associated with problematic smartphone use when compared to adults. Nevertheless, adolescents are more open to guidance for change. Undertaking such a study in Turkey is especially important since Turkey is classified as one of the countries with the highest risk potential of problematic smartphone use. Recent figures highlight that the percentage of Turkish users using WhatsApp heavily is 94% and Instagram with 80% is way higher than that of the European Union averages which is reported as 55% and 35%, respectively [32]. These rates place Turkey either to the first or the second place all over the world and above the averages of European countries.

In recent years, digital health related smartphone apps specifically designed to help individuals achieve positive behavior change started to emerge. These apps frequently use persuasive technology itself as a tool to reduce unhealthy, problematic smartphone use. The techniques used incorporate gamification, self-monitoring and goal setting [34].

However, to our knowledge, there is no structured education program that uses gamification, hook and the like techniques, in reverse manner, to decrease problematic smartphone use among adolescents.

### 1.1. New Marketing Strategies Appealing to the Unconscious 

The highly competitive modern marketing environment of the 21st century imposes unique challenges. Whether online or offline, the abundance of marketing messages coming from a variety of sources has caused consumers to have difficulty in devoting their time and attention to those messages. With the advent of the Internet of things (IoT) and big data, consumers currently witness an even higher level of digitalization, which takes the aforementioned phenomenon to a different level. 

In such a world, digital ecosystems are characterized by attention economy [35] in which marketers offer their services in exchange for the consumers’ attention. Monetization occurs indirectly through advertisers. Facebook, Instagram, YouTube, Twitter are typical examples which do not charge consumers but monetize their attention in this attention economy.

Since appealing to the conscious mind in the attention economy becomes increasingly difficult marketers have started to resort to alternative strategies that appeal rather to the unconscious. As such, digital platforms, particularly in the last decade, are in an arms race to grab and keep the consumers’ attention using sophisticated techniques in today’s always on and connected world. 

The flow theory, which has the aim of achieving the flow state, an emotional state embracing perceptual distortion and enjoyment [36] is one such strategy. Built on flow theory, new marketing strategies like gamification and user experience design which are collectively named as illusional marketing strategies [37,38] provide shortcuts to consumers by enabling them to take action without effortful thinking. The following sections provide further details on the marketing strategies, namely gamification and hook strategy, which make up the main focus of this paper, and are developed to design addictive experiences by appealing to the unconscious.

#### 1.1.1. Gamification 

Gamification can be described as the use of game design features in non-game contexts [39]. Through a gamified process, game elements and mechanics like achievements, progress bars, badges, leader boards, levels and points are employed to engage users [40]. In the context of marketing, gamification refers to a new marketing strategy that aims at changing the consumers’ behavior in predetermined ways to foster engagement and ultimately to buy products [41,42]. In order to attain this goal, designation of an engaging experience plays a key role. Previous studies show that gamified and engaging experiences may result in brand recall and buying behavior [43].

Marketing scholars classify gamification as a different kind of persuasive marketing [44], which raises more ethical concerns than other persuasive marketing strategies like product packaging, personalized digital marketing, etc. Some other scholars define gamification as a form of stealth marketing which refers to configuring a product in such a way that people are not aware that the marketer is trying to persuade them to buy it [45]. These concerns arise out of the degree and type of engagement between the brand and consumers due to the highly engaging gamified experience. Deep sense of participatory engagement in gamification can also be compared to co-creation marketing [46,47] in which consumers’ co-produce products by actively engaging in the design process [48]. However, gamification can have subversive results.

#### 1.1.2. Hook Strategy

Hook is a recent marketing strategy similar to gamification, based on behaviorist thinking related to marketing software products. It has its roots in Persuasive Technology Lab in Stanford University, founded by BJ Fogg, back in 1998. This relatively new field has been named ’captology’, which is an acronym for ’computers as persuasive technologies’. The ultimate goal in captology is to provide meaningful input into how software products can be designed to change consumer beliefs and behaviors [49].

Habitual behavior is central to such strategies. Habit-driven, automatic behaviors occupy the majority of the daily life and are performed with little, or no conscious thinking at all. Previous studies show that following a trigger-action-reward loop, a desirable habit can be imposed on an individual [50,51,52].

Impressed with Fogg’s work, Eyal [53] developed the Hook model, which is grounded on the reinforcement theory in forming habitual behavior, which goes back to reinforcement experiments [54]. The Hook model advises a four-step loop to make the desired behavior a habitual one. The first step is the trigger, which refers to an external or internal stimulus that tells the user what to do next and how to behave next. The second step, action, refers to the actions of the user based on the information provided by the stimulus. The third stage, namely reward, refers to the acquisition of a variable reward as a consequence of the actions mentioned above. The last step, investment, means having the users make a deliberate effort to invest in the platform as in the form of time, effort, social capital investment to increase the likelihood of the repetitive behavior.

The Hook model can be illustrated through a typical Instagram usage habit. The process starts with a notification message, which is an external trigger. The model aims at starting with an external trigger and gradually making it an internal one. Internal triggers are states of mind and emotions. For the behavior to be habitual, the emotion should be somewhat vague and negative like loneliness, boredom, uncertainty, etc. Vague emotions allow the desired behavior to become a habit by avoiding reaching the consciousness level while negative emotions cause the individual to take action more easily [28].

Action refers to the behavior of entering the Instagram application. The key in the action step is to make the desired behavior as effortless as possible. This is because the level of effort required in the behavior directly affects the habitual nature of the behavior [53]. In this context, instead of requiring the user to enter a password, smartphones now enable a mindless sort of authentication by fingerprint or retina scans. This allows consumers to take the action step literally in an effortless manner. Auto-activation of the smartphone camera by a single swipe and the similar features serve the same goal. 

The next step is the variable reward. It is what the notification is all about. For example, the user might have uploaded a photo album that consists of many photos. The variable reward is the discovery of which photo got the most likes, how many likes are received in total, who have commented and what they said about the posts and so on. The fact that the reward is variable allows the unconscious to be stimulated by surprise, causing dopamine release in the brain and regarding the perception of the reward as reinforcing [55]. Immediate variable rewards work perfectly well when the brain is in the unconscious mode [55] by gradually associating the reward with the trigger. 

Additionally, it has been documented that this state of mind causes an individual’s long run self to have no control over his or her decisions [56], which in turn causes a compulsive and addictive behavioral pattern.

The last step, investment to the platform, refers to invest in a small effort on the platform in such a way that calls for the repetition of the behavior over time, and finally makes it impossible to break off from the cycle. In Instagram application, this investment is the number of followers and the photo archive accumulated so far. The more investment is made in the platform, the more addictive it becomes. The system aims to replace the external trigger with the internal trigger in the long term. In Instagram application, the internal trigger is the desire not to miss the opportunity to capture a photo of the moment. An individual’s Spotify list, for example, is perceived as valuable since he/she has spent a considerable amount of effort creating that list. 

The addictive nature of various digital platforms was confirmed in various studies [31,57]. Some authors who investigated the gamification strategy also warn that the application of variable rewards may create gambling-like addictive experiences, which may lead to consumers being unable to exercise their free will [58]. The flow state is a state of mind characterized with enjoyment and perceptual distortion in time and space contributing to the addictive behavior [36]. The Hook model could be classified as a more sophisticated form of gamification since it targets the unconscious, applying gamification elements such as variable rewards and is designed to be addictive in nature. In this study, both hook and gamification strategies will be included in the education program considering their potential to create new habitual behavior [30].

Finally, this study first sought to explore the demographic factors influence on the adolescents’ problematic smartphone use, namely gender, age, grades and parental education [59,60,61,62]. Afterwards, the main focus of the study is to find out how does a marketing based education program reduces the problematic smartphone use of adolescents. Further, and last of all, the study investigates how the smartphone problematic use changed after the education according to the above stated demographic factors as gender, age, and grades except parents’ education. For all these research aims, the following hypotheses are formulated under three main categories, which are before the education program, about the education program and after the education program. Thus, the following hypotheses guided the present study. 

Before the education program;

**Hypothesis 1** **(H1).** *The problematic smartphone use differs according to demographic factors.*

**Hypothesis 1** **(H1a).** *The problematic smartphone use differs on the basis of gender.*

**Hypothesis 1** **(H1b).** *Age and problematic smartphone use are related.*

**Hypothesis 1** **(H1c).** *The problematic smartphone use differs on the basis of the adolescents’ grades.*

**Hypothesis 1** **(H1d).** *The problematic smartphone use of adolescents differs on the basis of their parents’ education level.*

About the education program;

**Hypothesis 2** **(H2).** *The adolescents’ problematic smartphone use will decrease after receiving a marketing based education program including gamification and unhook strategies.*

After the education program,

**Hypothesis 3** **(H3).** *The problematic smartphone use differs according to demographic factors after the education program.*

**Hypothesis 3** **(H3a).** *The problematic smartphone use decrease differs on the basis of gender after the education.*

**Hypothesis 3** **(H3b).** *Age and the problematic smartphone use after the education are related.*

**Hypothesis 3** **(H3c).** *The problematic smartphone use differs on the basis of the adolescents’ grades after the education.*

## 2. Method 

### 2.1. Participants

The sample consisted of 337 students from 14 different schools. Out of 337 participants, 32 were excluded since they were outliers or had missing data. In total, 144 girls (47.2%) and 161 boys (52.8%) participated in the study. Their mean age was 14.57 (SD = 0.74). 

### 2.2. Procedure

#### 2.2.1. Before the Education Program

The researchers have promoted the free education program to decrease problematic smartphone use on the social media platforms of the associated university. After that, the university corporate communications and public relations department e-mailed about the education program to school counselors of various schools residing in Istanbul. Following this, the education program was scheduled with the school counselors who requested it. Afterwards, school counselors have chosen the class sections randomly. Then, the school counselors sent to their families two weeks before the parental permission form including an information sheet about the research and a certificate of consent about the adolescent’s participation to the education program and research.

On the day of the education after the education, those students who volunteer to participate signed an assent form. If the adolescents did not sign the assent form they were not included in the study. Thus, only 337 out of 1055 students signed the assent form. Then, the researcher explained the procedures to the participants to complete the surveys. The participants were allowed to ask questions if they encountered problems while completing the questionnaires, and the research assistants helped them to resolve their problems.

#### 2.2.2. Description of the Education Program 

The intervention included a PowerPoint presentation accompanied by several striking videos that highlight the non-neutral characteristic of technology. The adolescents were also given flyers that highlight the key points as reminders of the education and a check-list of to-do items to be performed throughout the three-week observation period.

The whole program was delivered in one day using two breaks. The education program consisted of eight modules. Each module of the program roughly lasted about 15 min. The first module consisted of creating awareness about the problematic use of smartphones. The educator presented famous technology figures’ examples and introduced statistics about the problematic smartphone use. 

The module included evidence based striking facts and insights on how Silicon Valley-based technology platforms create addictive experiences. For example, Twitter, Instagram and Facebook platforms create a “craving” effect on users by making them wait (on variable time) to get their liking feedback. 

Once the participants were oriented and motivated to be a conscious smartphone user, then the next module was implemented, which showed how to measure and keep track of the participants’ screen time. The educator explained in detail the hook strategy that marketers employ to engage users. 

The next module was about presenting the participants how to unhook from their smartphones. Snapchat’s Snap Streaks feature could be regarded as a deceptive example to hook strategy. This feature shows the number of consecutive days someone has messaged to his or her respective friend as a badge. Adolescents see it as an indication of how strong their relationships are with their acquaintances and hence they do not want to break the streak. During the education program, some participants reflected by stating that when they were to be offline for a certain period of time (during a holiday with their family, etc.) they gave their user credentials to one of their close friends and asked them to send dummy messages to their stated friends so as not to break their snap streaks. 

In order to practice unhook strategies, the participants were first asked to identify the applications that made them feel unhappy. These applications were removed with the guidance of the educator. Additionally, in this module, the participants were persuaded to hide the social media applications that hooked them inside a folder. The home screen was cleared up and the time consuming applications were saved in another folder. 

During the third module, participants learned how to turn off all the notifications. Also the educator instructed to mute the group messages and to remove the last seen or read information from the account menu under the privacy settings. Once all the sources of distractions were removed, the fourth module was initiated. 

It was in the fourth module that a digital diet program was introduced. The fifth module integrated mindfulness applications as well as attention focusing techniques to control unconscious use. The sixth module included strategies to increase the quality of the participants’ social relationships. The adverse effects of being available all the time and its damaging impact on personal productivity were explained along with supportive research findings. Afterwards, steps that could be taken to prevent the problem were introduced. On WhatsApp, for example, the adolescents were advised to avoid immediate reply to not-so-urgent incoming messages and to put a deliberate delay so that the other party’s anticipation for an immediate reply was lowered. Then, the educator taught the participants to build the communication rules and facilitate the quality of social communication without phone. Recent studies showed that 72% of adolescents (as opposed to 48% of adults) feel an urge to respond to an incoming call or a message immediately [63]. This phenomenon can be attributed to the wrong interpersonal communication etiquette as well as low self-control and peer pressure. Therefore, the education module also incorporated the proper etiquette for communication that relieves the individual from feeling the urge to respond to everything immediately.

All through the seventh module a gamification strategy was used to engage the adolescents with the use of #iamincontrol hashtag use. This module of the education was about adolescents’ controlling their lives. The participants learned how to protect an area they identified as a phone-free zone. The educator taught when, where and how to leave their phone in their daily routines. 

The participants were also offered many activities they could plan together with their loved ones. Along the modules, the participants were asked to do homework and were supported with manuals, digital diet planners, activities list and handy calendars. Once all the content was delivered, the researcher scheduled a follow-up meeting three weeks after with the same school. 

#### 2.2.3. After the Education Program

Three weeks later, the scales and checklist were collected from the participants with the help of the school counselors. The participants who conducted the demographic questionnaires and the smartphone addiction scale before the education were distributed an id number so that it matched the collected surveys after the program. The researchers prepared an evaluation form to test the effectiveness of each task in the education program. Then, the participants were asked to rate about the effectiveness of each step in the program. Finally, they rated these steps according to their own usage benefits.

### 2.3. Measures

Demographic Form: The form includes gender, age, parents’ education, parents’ occupation, social media use of the family and the adolescent, purpose of use for the smartphone and the time they started using it and how much time they spent on it on a daily basis. 

#### The Smartphone Addiction Scale for Adolescents 

The Smartphone Addiction Scale for Adolescents (SASA) measures smartphone addiction. SASA was developed by Kwon et al. [60]. The Turkish validation study conducted by Demirci et al. (2014) consisted of 6 point Likert scales ranging from 1 (Definitely No) to 6 (Definitely Yes), 36-items and 7 factors as disturbing daily life and tolerance, withdrawal symptoms, positive anticipation, overuse, social network dependency, physical symptoms and cyberspace-oriented relationships [61].

The correlations between the subscales ranged from 0.22 to 0.51 The highest total scales values indicated a higher risk for problematic smartphone addiction. The Cronbach’s α’s of the seven factors were found respectively as 0.85, 0.81, 0.74, 0.62, 0.59, 0.64, 0.34 and 0.89 for the total scale in this study, which are very close to the validated scale. The Pearson correlations coefficients varied between the SASA factors and total varied between 0.18 and 0.75, indicating statistically significant positive relations (all *p’s* < 0.01). Since it is a reliable and valid tool of measurement, SASA was conducted to assess the change in the smartphone usage before and after the education program. 

### 2.4. Statistical Analysis

The data analysis was conducted using SPSS 21 for Windows. Descriptive statistics were calculated to investigate the demographic data. In order to test first and third set of hypotheses, the demographics of the participants were compared by independent sample *t*-tests analysis of variance tests. Pearson moment correlation analysis was run to evaluate the relationships between continuous demographics (e.g., age) and the factors of SASA. In order to test Hypothesis 2 which aims to evaluate of the effectiveness of education, paired sample *t*-test was used to compare before and after SASA scores.

#### 2.4.1. Demographic Results

Three hundred and five participants were included in this study. 144 (47.2%) were female, and 161 (52.8%) were male. Only 44 (14.4%) of the participants indicated that they had cyber friendship, which they had met online and then later got to know in person. Table 1 listed gender, grades, electronics they possess, purpose of use, applications they have and parents’ education. 

#### 2.4.2. Difference Tests Results before the Education 

Smartphone use variable was the reported use of smartphone time as minutes per day (X_use_ = 139.84, SD = 83.68). The daily use of smartphones was declared to be less than 180 minutes for 75%, between 180 to 300 min for 21.5% and more than 300 min for 3.5% of the sample. 

The first set of hypotheses explored the problematic smartphone use difference in terms of gender (H1a), age (H1b), adolescents’ grades (H1c) and parents’ education (H1d). In order to test gender differences, an independent sample *t*-test was conducted. As a result, there were no significant difference between boys (X_boys_ = 81.06) and girls (X_girls_ = 84.99) in terms of total SASA before the education (*p* > 0.05). 

Then, to test the age and SASA relation (H1b), a Pearson moment correlation analysis was conducted. The findings revealed no significant relationship between age and SASA scores (r = 0.01, *p* > 0.05). 

In order to test the problematic smartphone use difference on the basis of the adolescents’ grades (H1c) an analysis of variance test showed that SASA scores before the education do differ significantly according to the students’ grade levels. A Tukey post hoc test indicated that the SASA scores increased significantly at 10th grade (X_Pep_ = 16.19 X_9th_ = 17.26 X_10th_ = 19.33 X_11th_ 17.06 *F*_(305)_ = 3.59, *p* < 0.05 d = 0.3). 

In order to measure the problematic smartphone difference on the basis of the parents’ education (H1d) an analysis of variance test indicated that the adolescents’ SASA scores vary according to their mothers’ education level. A Tukey post hoc test showed that there was a significant difference between high school graduates and college graduates’ mothers in terms of their children’s SASA scores (X_MiddleSchool_ = 17.39 X_HighSchool_ = 18.13 X_College_ = 16.52; *F*_(305)_ = 3.62, *p* < 0.05). Another analysis of variance test revealed that fathers’ education also differed in terms of their children’s SASA scores. A Tukey post hoc test showed that there was a significant difference between high school graduates and college graduates fathers’ in terms of their children’s SASA scores (X_MiddleSchool_ = 16.95 X _HighSchool_ = 18.22 X_College_ = 16.89; *F*_(305)_ = 3.21, *p* < 0.05 d = 0.3). 

#### 2.4.3. Difference Tests Results after the Education

In order to test the second hypothesis, which evaluated the influence of the education program, a paired sample *t*-test analysis before and after the education program was conducted. As shown in Table 2, the results revealed a significant decrease in disturbing daily life and tolerance (X_pretest_ = 2.93 X_posttest_ = 2.68), withdrawal symptoms (X_pretest_ = 2.09 X_posttest_ = 1.97), positive anticipation (X_pretest_ = 3.18 X_posttest_ = 2.84), overuse (X_pretest_ = 2.61 X_posttest_ = 2.44), physical symptoms (X_pretest_ = 2.67 X_posttest_ = 2.51) dimensions and in the total SASA scale (X_pretest_ = 17.30 X_posttest_ = 16.19). Cyberspace oriented relationships and social network dependence factors did not change significantly after the education program (*p* ‘s > 0.05). Thus, the effectiveness of the education program has been proven for total and five factors except cyberspace oriented relationships and social network dependence factors as can be seen in Table 2.

In order to test the third set of hypotheses that explore the difference of the problematic smartphone use in terms of gender (H3a), age (H3b) and adolescents’ grades (H3c). Firstly, an independent sample *t*-test was conducted to compare boys’ and girls’ SASA scores after the education program (H3a). The results revealed that male adolescents’ total SASA dropped significantly more than those of the girls with a medium effect size (X_female_ = 17.17 X_male_ = 15.31; *t*_(305)_ = 3.63, *p* < 0.01 d = 0.42). Additionally, the SASA scores of the participants after the education have been subtracted from the SASA scores before the education. Boys’ change is significantly higher than that of girls’ change (X_girls_ = −0.58 X_boys_ = −1.58; *t*_(305)_ = 2.55, *p* < 0.01 d = 0.30). 

Following this, to test the age and SASA relation after the education program (H3b), a Pearson moment correlation analysis was conducted. The findings revealed no significant relationship between age and SASA scores after the education program (r = −0.01, *p* > 0.05). 

After the completion of the education, an analysis of variance test indicated that SASA scores varied significantly according to the adolescent’s grades. A Tukey post hoc test showed that only the SASA scores of preparation grade and 10th graders were significantly different (X_Pep_ = 14.56 X_9th_ = 16.36 X_10th_ = 17.33 X_11th =_ 16.23; *F*_(305)_ = 2.66, *p* < 0.05 d = 0.02). 

### 2.5. Feedback Results about the Program

The percentage of the participants who practice no-tolerance rule to phubbing friends, those who prefer to look to their smartphone screens instead of themselves in a physical conversation were 62% and 56% of them have found this method to be somewhat or absolutely useful. The corresponding percentages for turning-off all notifications (except from real people) were 55% and 58%, putting all WhatsApp group messages to silent mode were 68% and 65% and turning off the last seen time and read receipt of WhatsApp messages were 58% and 56% respectively.

Initially, many adolescents had reported that they had hard time trying them. After experiencing these methods, however, many of them reflected that they feel more in control of technology and can now focus more easily on their tasks. 

## 3. Discussion

This study first aims to investigate the role of demographic factors, namely gender, age, grades and parents’ education level of adolescents in assessing their tendency for smartphone addiction before the education. The first hypothesis related to demographics’ differentiation in terms of problematic smartphone use is examined separately for gender, age, grades and parental education. The problematic smartphone use did not differ on the basis of gender nor has been associated with age. Only the problematic smartphone use differs for grades and parental education. The second hypothesis, which constitutes the main objective of this study, is about inquiring the effectiveness of a novel education program for reducing problematic smartphone use in adolescents. In order to achieve this, a marketing based education program has been developed and then later measured the effect before and three weeks after the program. The change in problematic smartphone use showed a significant decrease. Moreover, the last hypothesis is related to the demographic factors, specifically gender, age and grades. These demographics are critical in order to understand which ones differed after the education for future references. Gender and grades differed in terms of decreases in problematic smartphone addiction. 

Concerning the first hypothesis, the current study revealed that there is no statistically significant difference between girls and boys in terms of their SASA scores before the education. Thus, the first hypothesis of this study is rejected confirming the previous studies [59,60]. There are many studies that indicated female addiction is higher than males [64,65,66]. These studies attribute females’ higher tendency for mobile addiction to females’ being more likely to engage in interpersonal communication and social relationships via their mobile phone [67,68]. Moreover, Demirci et al.’s (2014) study conducted in Turkey indicated higher SASA scores for female participants. The fact that the difference between boys and girls is not significant in this present study can be explained first by age factor. The participants of this study consisted of adolescents while Demirci et al.’s (2014) study consisted of college students. Second, not only the age group might have brought out the discrepancy between these studies but also the six years’ difference (2014–2020) might have changed the rates. Indeed, gender gap is predicted to decrease over the years with the advances in technology spreads, which also explains the current gender similarity in terms of addiction [69]. The total SASA scores of the adolescents in this study (girls were 84.99 and boys were 81.06) are higher but the gender difference has closed compared to previous findings [60] as (females were 78.7 and males were 72.2). As a matter of fact, this finding confirm that the gender gap is closing and problematic smartphone use has gradually become more prevalent among adolescents in recent years [32]. 

This study could not find a statistically significant relationship between age and problematic smartphone use. As Demirci et al. (2014) study [60] also found non-significant negative relationship between age and smartphone addiction, the results are in line with the previous study. Some studies, particularly in the Internet addiction domain, find such negative but significant relation between adolescents’ age and addiction level stating that this risky behavior is temporary in the adolescence period. This phenomenon is said to wear off with increased familiarity with the technology [70,71]. 

The problematic smartphone use differed significantly on the basis of the adolescents’ grades. Beginning around age 10 adolescent risk taking, reward sensitivity behaviors start increasing, then peak between 13 and 16, and then decline [72,73,74]. The problematic smartphone use according to grades is in line with the risk preference trend. The earlier grades’ SASA scores are lower than the later ones, then there is a peak at 10th graders and then it drops again. 

The higher the maternal and paternal education level the lower were the SASA scores except for college graduates. The college graduate parents are mostly white collar workers who might spend less time and control over the adolescents which might have caused more problematic smartphone use. Thus, this finding of the present study partially confirms the previous studies in the literature [66,67]. In the light of these findings, we can conclude that, both parents’ education and attention, which ultimately contributes to a more beneficial attitude towards adolescents [66]. 

The main aim this study is to reduce the problematic smartphone use after a marketing based education program. The education program started with explaining the negative consequences of problematic smartphone use in terms of its harm on cognitive capacity, academic performance and social relationships. Afterwards, the internal mechanics of addictive design elements of digital products are explained in detail. The program followed by going through the steps in the hook model aimed at exercising the opposite behavior in each step to prevent unconscious, compulsive smartphone usage and ensure conscious and healthy usage behavior. The education program was coupled with a follow-up meeting three weeks after the education. The education module aimed at building awareness on the specifics of addictive mechanisms that ultimately hook users. 

Concerning the second hypothesis, the results are very promising since the comparison of SASA scores before and after the education program has proven significant decrease in problematic smartphone use. The disturbing daily life and tolerance, withdrawal symptoms, positive anticipation, overuse, physical symptoms dimensions and the total SASA scores decreased significantly after the education program. The cyberspace-oriented relationships and social network dependence factors did not change significantly after the education program. When the cyberspace-oriented relationship factors’ questions items are checked closely, there is a distinction between cyber friends and friends. Only a small percent of the participants did make cyber friendship (14.1%). This fact might be due to the more conservative nature of families in Turkey [75] in which adolescents are still protected closely. Finally, the social network dependence factor did not change significantly after the education. The two items that measured social dependence did include social applications as Facebook and Twitter. The participants’ reports indicated that the rate of use for Facebook (24.6%) and Twitter (18.5%) is considerably low. Since most of the participants do not use these applications, these questions might have misdirected them. 

Concerning the third hypothesis, the boys’ SASA scores decreased more compared to those of the girls. The addiction literature provides evidence of that women had 2.7 more risk at smartphone addiction than men [76]. When women try to be abstinent, they show greater anxiety and stress compared to men [77,78]. First, it might be less stressful to reduce the problematic smartphone use for boys than girls. Furthermore, girls tend to strengthen their friendships, which keeps them on the smartphones and receive more emotional support, while boys aim to use smartphones to extend their weak social relations [79]. The education program’s emphasis on how to build social relationship without the use of smartphones might have reduced boys’ problematic smartphone use more than the girls. Girls define smartphones as central component of their personal existence [80] which could bring more resistance to reduce their smartphone use. 

It was found that there is no significant relationship with adolescents’ age and smartphone addiction levels after the education program. There are in the literature longitudinal and cross sectional studies where the expected behavior and age were not correlated after the intervention either [81,82]. A meta-analysis about the intervention programs applied to subjects under 18 years of age revealed that age did not have an influence on the success of the programs [83]. However, the later study mentioned about the possibility of cognitive developmental level, which might be related to grades. 

The problematic smartphone use differed significantly on the basis of the adolescents’ grades after the education. As a matter of fact, this finding pattern is very similar to the case before education. This illustrates that adolescents at earlier grades are still more open to guidance for healthier and more proper communication suggestions. This can be also explained by the fact that the education could be more influential when the habitual behavior of using a smartphone has recently started. 

The results also reveal that, with a 97.2% usage rate, smartphone use is much more prevalent than the use of any other technology products. The results also show that among the responding adolescents, 57.6% check their social media from their smartphone more than 10 times a day. WhatsApp was found to be the most frequently installed mobile app; almost all the responding adolescents (98.2%) use it. Instagram (80.5%) was found to be the second mostly used app among the adolescents.

Overall, the results of the study suggest that educational guidance for healthy usage of technology is missing or not adequate in the visited schools. Unfortunately, the current educational system fails to provide even the basic communication etiquette to adolescents. That is, adolescents do not know and are eager to learn the proper way to communicate with their peers (importance of face to face communication etc.). This is especially valid for younger adults as opposed to their older counterparts.

### Limitations and Implications for Future Research

Due to the peculiar characteristics of this special age group, namely adolescents, self-reported inventories like the one in this study have self-assessment bias risk. The results may also differ in terms of participants’ self-involvement level, sincerity, beliefs etc. In addition, this study has been conducted in a relatively more conservative culture, which might have driven participants to give desirable answers. 

All in all, the program is distinguished in that an improvement is achieved using a structured program. However, in the future, longer and more thorough educational programs could be designed to achieve more lasting effect. In addition, such an educational program could have much more impact if coupled with a digital health app usage that tracks individual behavior.

## 4. Conclusions

Technology platforms have been creating increasingly more addictive experiences that result in spending prolonged periods of time on screens, which ultimately leads to more profitability in the attention economy. Some of these addictive features include the lack of stop sign facilitated by infinite scroll feature, the hooking technique that relies mainly on the unconscious and employs mechanisms like variable rewards. 

Problematic smartphone use increasingly becomes more of a concern globally especially among adolescents. In this study, a marketing approach was adopted in an education program. Consumer-engaging methodologies are used to increase awareness on specifics of hooking strategies and to disengage adolescents from their phones. The results are significant in that an apparent improvement in problematic smartphone use is accomplished with a short and structured education program. Although there are some mindfulness-based intervention studies that have proven to reduce smartphone use [84], to our knowledge, this is the first study in which a marketing-focused education program has been developed to approach smartphone addiction. The program also offered practical, ready-to-use tips to restrain oneself from unconscious, compulsive smartphone use. 

This study makes significant contributions to the existing literature in several aspects. Firstly, looking from a marketing perspective, it uses the strategies employed to increase compulsive, unconscious smartphone usage, but in reverse direction to prevent excessive and hence problematic smartphone use. In addition, an educational program was introduced with a special emphasis placed on the conscious usage of smartphones. 

Living without digital technologies has become almost impossible. This is particularly true as global pandemics (i.e., Covid-19 virus a.k.a. Corona virus) and the like developments are poised to change our daily habits radically. However, the key question in our relationship with technology is about which party will be in control. Unconscious, compulsive smartphone usage lets technology control users. It will be only by educating the society with the proper way of smartphone use that we can turn the situation the other way around. 

The conducted study was scheduled as a one-time education. However, if these eight modules could be spread across the eight-week period with more supportive homework and with more detailed monitoring, the benefits could be improved. Future studies could integrate cognitive behavioral approaches and more gamified activities in order to hook adolescents back to their own future.

## Figures and Tables

**Table 1:** Demographics of the Participants.

N = 305	Frequency	Percentage
**Gender**		
Female	144	47.2
Male	161	51.8
**Grades**		
Prep (14 yrs)	44	14.4
9th Grade (15 yrs)	210	68.9
10th Grade (16 yrs)	31	10.2
11th Grade (17 yrs)	20	6.6
**The distribution of electronics they do possess**		
Smartphone	305	100
Computer	187	61.3
Pad	82	26.9
Console	57	18.7
**Smartphone purpose of use**		
Education/Research	275	90.2
Entertainment/Game	239	78.4
Free Time	207	67.9
Social Media	238	78.0
Communication	217	71.1
Shopping	124	40.7
**Applications participants have**		
WhatsApp	302	99.0
Instagram	247	81.0
Facebook	75	24.6
TicToc	10	3.3
Snapchat	102	33.4
Twitter	58	19.0
Pinterest	67	22.0
YouTube	222	72.8
Twitch	43	14.1
**Mother’s Education**		
Middle School	125	41.0
High School	78	25.6
College	102	33.4
**Father’s Education**		
Middle School	89	29.2
High School	91	29.8
College	125	41.0

**Table 2:** Paired sample *t* test results before and after the education program.

Factors	Mean Difference	St. Dev	*t*	*p*
1. Disturbing daily life and tolerance	0.25	0.81	5.29	<0.01 ^**^
2. Withdrawal symptoms	0.12	0.75	2.56	<0.01 ^**^
3. Positive anticipation	0.34	0.88	6.73	<0.01 ^**^
4. Cyberspace oriented relationships	0.03	0.74	0.75	0.45
5. Overuse	0.17	0.89	3.32	<0.01 ^**^
6. Social network dependence	0.06	1.05	1.06	0.29
7. Physical symptoms	0.15	0.89	2.97	<0.01 ^**^
Total SASA	1.11	3.45	5.61	<0.01 ^**^

Notes: ** deemed significant at the 0.01 level.
